# Dr. Weston D. Bidaman

**Published:** 1907-03

**Authors:** 


### OBITUARY.

DR. WESTON D. BIDAMAN, of Buffalo, died at his home February 15, 1907, after an illness of several months, heart trouble being announced as the cause of death, aged 55 years. He was born in this city and graduated in medicine from the University of Buffalo in 1878. Dr. Bidaman was in continuously active practice from the date of graduation until he was seized with the illness that proved fatal—a period of nearly thirty years. His professional life, especially in later years, had been a busy one and he obtained and retained the respect of his colleagues, as well as that of a large acquaintance. He was a member of the Medical Society of the County of Erie. Dr. Bidaman is survived by his wife, Marie L. Bidaman, nee Barrows, and one son, Miner D. Bidaman. The funeral was held from the family home on Sunday afternoon, February 17, at 2:30 o'clock, and the interment was at Forest Lawn.

